# Beyond Reperfusion: Early Molecular Drivers and Therapeutic Opportunities in Acute Post-Infarction Cardiac Fibrosis

**DOI:** 10.3390/ijms27104409

**Published:** 2026-05-15

**Authors:** Desaree Tan, Yi Ee Lye, Pranjal Patel, Nay Aung Minn, Anne Cao Le, Alex Bobik, Tin Kyaw

**Affiliations:** 1 Inflammation and Cardiovascular Disease Laboratory, Baker Heart and Diabetes Institute, Melbourne, VIC 3004, Australia; desaree.z.tan@gmail.com (D.T.); yiee.lye@baker.edu.au (Y.E.L.); patel.p@student.unimelb.edu.au (P.P.); nayaungminn@gmail.com (N.A.M.); anne.le@baker.edu.au (A.C.L.); alex.bobik@baker.edu.au (A.B.); 2 Centre for Inflammatory Diseases, Department of Medicine, Monash University, Clayton, VIC 3168, Australia; 3 Department of Immunology and Pathology, Monash University, Melbourne, VIC 3168, Australia

**Keywords:** cardiac fibrosis, inflammation, cell death, myocardial infarction, death signalling, heart failure

## Abstract

Heart failure is a leading cause of global morbidity and mortality, often developing as a consequence of acute myocardial infarction. Current management focuses on timely reperfusion via percutaneous coronary intervention. Yet, this approach fails to prevent the molecular cascades that drive the death of viable yet stressed cardiomyocytes within the infarct and peri-infarct zone. Effective antifibrotic therapies remain limited, highlighting a critical gap in current management strategies. This review aims to integrate current understanding of the molecular mechanisms underpinning post-infarct fibrosis and potential interventions for therapeutic development. This emphasis on molecular death signal activation and cell elimination highlights the redundancy of interconnecting fibrosis pathways. Anti-inflammatory and cell-targeted therapies focussing on oxidative stress and haemodynamic load have demonstrated strong preclinical promise. Yet, these approaches have largely failed to translate into clinical benefit. Overall, these limitations emphasise a narrow therapeutic window for intervention. As such, current therapies often fail to preserve metabolically vulnerable myocardium that remains potentially salvageable. Therefore, emerging approaches including RNA-based therapies, cardiac reprogramming, and targeted delivery systems offer new opportunities to improve therapeutic precision. Collectively, these findings support a shift toward early, cell-targeted intervention strategies. This approach aims to prevent progression to heart failure and increases patient quality of life.

## 1. Introduction

Heart failure (HF) is a leading cause of mortality, affecting 64 million individuals, with a median survival of five years [1]. Acute myocardial infarction (AMI) remains a prominent aetiology of HF, manifesting in 20–25% of patients post AMI [1]. Often marked by the presence of coronary artery stenosis, MI injury is driven by cardiomyocyte ischemia [2]. Impaired myocardial perfusion drives irreversible cardiomyocyte injury, fibrotic remodelling, and contractile dysfunction [3]. Such pathological changes can precipitate the development of malignant cardiac arrhythmias and HF [4]. Therefore, consequent sequelae of AMI and HF are associated with a decline in quality of life and impose a significant socioeconomic burden on the patient [5].

The risk of HF is influenced by the duration of myocardial ischemia and is closely correlated with the size of the infarct core and the peri-infarct zone [6]. A 5% increase in infarct size confers a 20% risk of developing HF [6]. Critically, patients diagnosed with HF following AMI exhibit a median survival of 3.4 years, emphasising HF burden on patient quality of life [7]. Currently, the gold standard for AMI management centres on percutaneous coronary intervention (PCI) within 90 min of presentation or fibrinolytics if PCI is unavailable within 120 min [8]. Yet, this approach may exacerbate myocardial injury through oxidative stress and downstream inflammation [9]. Critically, reperfusion injury is estimated to contribute to approximately 50% of the total myocardial infarct size [10].

Beyond restoring coronary perfusion, therapies are needed to preserve viable, metabolically stressed peri-infarct cardiomyocytes. Given the acute nature of AMI injury, development of interventions must centre on an understanding of the molecular mechanisms behind acute cardiac fibrosis. However, the causative mechanisms have not yet been fully understood. To mitigate HF, this narrative review will examine key pathways of cardiac fibrosis alongside existing therapeutic interventions. Collectively, this analysis will drive the identification of timely therapeutic targets in urgent AMI settings.

## 2. AMI Management and Reperfusion Therapy

Myocardial reperfusion is the first-line approach in AMI management [8]. Physiologically, the optimal time for mitigating cardiomyocyte injury is 15 min after infarct [11]. Following accelerated ATP depletion and lactate accumulation, contractile dysfunction ensues, reducing the viability of the cardiac muscle [11]. Existing guidelines champion PCI within 90 min after medical contact. Yet, catheterization and procedural logistics can extend door-to-balloon times, delaying optimal reperfusion [12]. However, when achieved promptly, successful reperfusion allows for immediate cessation of cardiomyocyte necrosis. A reduction in occlusion time mitigates electrical instability and mechanical complications, and stenting prevents reinfarction [13]. Evidently, the success of PCI hinges on the time to procedure.

Despite significant clinical benefits following PCI, reperfusion injury paradoxically remains a barrier to re-establishing full cardiomyocyte function. Reperfusion injury can account for 50% of the final infarct size, following initial infarct assault [10]. However, the mechanisms behind PCI injury are not yet fully understood. Of note, rapid restoration of blood flow is purported to increase the production of reactive oxygen species (ROS), promote complement activation and trigger microvascular endothelial dysfunction [14]. As such, stressed peri-infarct cardiomyocytes undergo coagulative necrosis [14]. Another purported mechanism is the no-flow phenomenon, where reperfusion fails to restore adequate microvascular circulation despite successful epicardial reperfusion [15]. Persistent microvascular ischemia thereby extends injury within the infarct zone and limits myocardial salvage [15]. Collectively, a predominant therapeutic focus on the infarct core has contributed to maladaptive ventricular remodelling and impaired contractile function.

**Figure 1:**
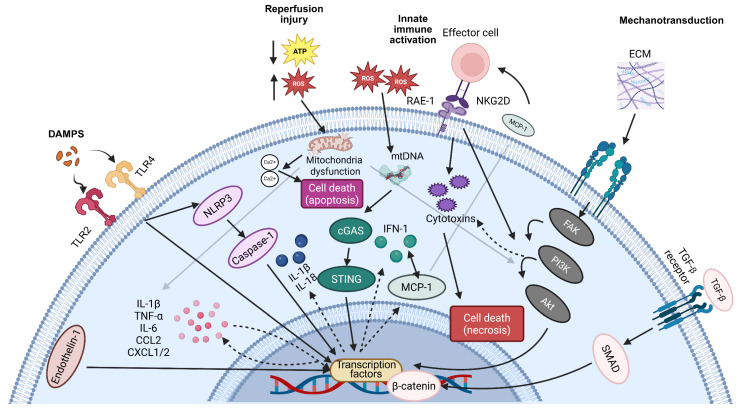
Pathophysiology of Acute Cardiac Fibrosis. Reperfusion injury following acute myocardial infarct triggers oxidative stress, mitochondrial dysfunction, and innate immune activation. Together, this initial insult promotes cardiomyocyte death and acute fibrotic remodelling. Excess reactive oxygen species (ROS), ATP depletion, and Ca^2+^ overload destabilise mitochondria. Critically, release of DAMPs (damage-associated molecular patterns) such as mitochondrial DNA, HMGB1, S100A8/A9 and HSPs activate toll-like receptors (TLR2 and TLR4). Downstream activation of the cGAS-STING pathway perpetuates type I interferon signalling and monocyte recruitment with MCP-1. Concurrently, DAMPs activate the NLRP3 inflammasome, further amplifying pro-inflammatory cytokine release via activation of transcription factor NF-κB and YAP/TAZ. Stress-induced expression of retinoic acid early inducible-1 (RAE-1) promotes engagement with NKG2D receptors on cytotoxic lymphocytes, triggering perforin- and granzyme-mediated cardiomyocyte necrosis. Involvement of TGF-β and Wnt pathways collectively mediates cytokine production alongside acute cardiac fibrosis. Collectively, each mechanism interconnects and contributes to acute cardiac fibrosis. Solid arrows and colour coding indicate pathway progression, while dotted arrows represent upregulated production or enhanced activation activation. Additionally, grey arrows display an indirect or secondary pathway interactions.

Mechanistically, reperfusion injury is driven by a coordinated cascade of oxidative stress, calcium overload, mitochondrial dysfunction and innate immune activation [14]. Sudden restoration of oxygen delivery through reperfusion may promote excessive mitochondrial production of reactive oxygen species (ROS), collapse of membrane potential, and ATP depletion within vulnerable peri-infarct cardiomyocytes (Figure 1) [11]. With mitochondrial instability, cytosolic Ca^2+^ overload further exacerbates cellular proteolysis, accelerating cardiomyocyte necrosis and the release of mitochondrial DNA [11]. Further to this, complement and endothelial activation contribute to local microvascular obstruction, creating the no-flow phenomenon following reperfusion [15]. Overall, this perpetuates local ischemia despite successful reperfusion, further expanding myocardial damage. With cell death, downstream activation of the NLR family pyrin domain-containing 3 (NLRP3) inflammasome and nuclear factor-κB (NF-κB) pathway drives neutrophil recruitment [16,17]. Collectively, these processes extend cardiomyocyte loss and initiate profibrotic remodelling despite successful reperfusion.

Efforts to mitigate reperfusion injury have focused on combination therapy inclusive of ischemic preconditioning and pharmaceutical methods such as N-acetylcysteine, glucagon-like peptide-1 (GLP-1) agonist (Exenatide) and cyclosporine A [14,18,19]. Ischemic preconditioning aims to increase tolerance of the myocardium to prolonged ischemic reperfusion injury. Use of intermittent limb cuff inflation and brief coronary artery occlusion promotes the cardioprotective accumulation of adenosine, bradykinin and protein kinase C (PKC) [19]. Pharmaceutical approaches aim to reduce ROS accumulation and promote cardioprotective signalling through the phosphoinositide 3-kinase-protein kinase B pathway (PI3K/AKT) [14,18]. However, each approach has produced non-significant reductions in mitigating reperfusion injury. Therefore, future interventions must focus on reducing infarct size and limiting early fibrotic remodelling to halt progression to HF.

## 3. Pathophysiology of Acute Cardiac Fibrosis in Myocardial Ischemia

Following an ischemic infarct, the rapid accumulation of metabolic by-products accelerates cardiomyocyte injury. Myocardial cell death initiates excessive deposition of extracellular matrix (ECM) proteins, predominantly collagen types I and III [20]. While this fibrotic tissue preserves ventricular wall integrity, its non-contractile nature impairs cardiac function [21]. Beyond the infarct core, sustained mechanical stress, neurohormonal activation, and inflammatory signalling promote rapidly reactive fibrosis in the peri-infarct zone [22]. Consequently, the expansion of fibrotic tissue increases myocardial stiffness, impairing both systolic and diastolic function and predisposing to HF and malignant arrhythmias [23].

Although the precise cellular mechanisms of acute cardiac fibrosis are not yet fully understood, the rapid surge in ROS and downstream inflammatory signalling emphasises the critical need for early therapeutic intervention (Figure 1). Elucidation of these pathways is essential to inform the development of targeted therapies aimed at limiting fibrosis and protecting cardiomyocytes.

### 3.1. Initiation of Death Signals: The Innate Immune System

Following myocardial ischemia, necrotic cardiomyocytes passively release damage-associated molecular patterns (DAMPs), which act to activate the innate immune system [24]. Key DAMPs liberated in infarcted tissue and activated neutrophils include Heat Shock Proteins (HSPs), high-mobility group box 1 (HMGB1), mitochondrial DNA (mtDNA), adenosine triphosphate (ATP) and calprotectin (S100A8/A9). These molecules engage pattern recognition receptors (PRRs), which are expressed on resident immune cells and cardiac fibroblasts. As a result, inflammatory signalling cascades rapidly evolve within hours of infarction [16,17,25].

Among the PRRs, TLR4 and TLR2 are prominent mediators of post-infarct acute inflammation and fibrosis. Downstream activation of canonical NF-κB signalling drives the rapid inflammatory release of IL-1β, TNF-α, IL-6, CCL2 and CXCL1/2 [26]. Critically, genetic deletion and pharmacological blockade of TLR4 are significantly correlated with reduction in infarct size, pro-inflammatory signalling and cytokine-release in ischemia–reperfusion injury models (I/R models) [27,28]. Furthermore, elevated TLR4 expression on circulating mononuclear cells has been observed in patients following AMI, highlighting the importance of systemic innate immune activation in early MI [29]. Following TLR engagement, extracellular ATP and mitochondrial DNA promote activation of the NLRP3 inflammasome. This results in downstream cleavage of pro-caspase-1 into pro-IL-1β and pro-IL-18, amplifying the inflammatory cascade [30,31]. Consistent with this mechanism, inhibition of the purinergic receptor P2X7 disrupts inflammasome assembly. This intervention attenuates infarct expansion and limits post-infarction cardiac remodelling [31]. Evidently, prevention of DAMP accumulation and activation of the inflammasome remain critical pathways in managing acute cardiac fibrosis.

This is further exemplified in the role of S100A8/A9 as a key mediator of early post-infarct cardiac inflammation [25]. Following AMI, the release of S100A8/A9 from activated neutrophils activates TLR4, crucially amplifying downstream canonical nuclear factor-κB (NF-κB) signalling. The upregulation of pro-inflammatory cytokines such as TNF-α, IL-6 and IL-17, promotes the recruitment of monocytes [25]. This perpetuates cardiomyocyte stress and injury within the peri-infarct border zone. Critically, experimental blockade of S100A9 increased left ventricular ejection fraction (LVEF) by approximately 13% in preclinical models following AMI [32]. Elevated circulating S100A8/A9 levels in post-AMI patients predicted an increase in adverse ventricular remodelling, heightening long-term HF risk [33]. This further emphasises the importance of mitigating early DAMP accumulation and the subsequent activation of PRRs.

Another proposed death signal initiation pathway focuses on the cyclic GMP-AMP synthase (cGAS)–Stimulator of Interferon Genes (STING) pathway. Following AMI, oxidative stress amplifies the production of ROS, destabilising the mitochondrial membrane. With the rapid accumulation of cytoplasmic mtDNA, activation of the cGAS-STING pathway drives a pro-inflammatory state with the release of type 1 interferons and monocyte chemoattractant protein-1 (MCP-1) [34,35]. MCP-1 facilitates immune cell trafficking to the infarct zone. In contrast, type 1 interferons interact with the interferon-α and β receptor subunit (IFNAR) in an autocrine fashion [36]. These acute stress signals induce the upregulation of Retinoic Acid Early Inducible-1 (RAE-1), a death signal which is minimally expressed in healthy physiological conditions [37]. The engagement of RAE-1 and the Natural Killer Group 2, member D (NKG2D) receptor expressed on Natural Killer (NK) cells, CD8^+^ T cells, γδ T cells, and NKT cells, releases perforin and granzyme B [38,39]. Cardiomyocytes thus undergo coagulative necrosis, perpetuating later fibrotic remodelling [22]. Antibody-mediated inhibition of the RAE-1–NKG2D axis in murine myocardial infarction models significantly reduced cardiomyocyte death and cardiac fibrosis, thus mitigating progression to HF [40]. Critically, this approach preserved left ventricular systolic function emphasising the role of RAE-1 stress-ligand signalling as a driver of post-infarct remodelling [40]. Therefore, the prevention of cardiac fibrosis centres on halting the acute-phase accumulation of cytoplasmic mtDNA. Reducing early cardiomyocyte stress and repair must be the focus in mitigating cardiac fibrosis.

### 3.2. Mechanotransduction

Following ischemia, early ECM remodelling generates pathological mechanical signals triggering aberrant cardiac fibrosis. Rapid upregulation of matrix metalloproteinases (MMPs), particularly MMP-2 and MMP-9, facilitates fibroblast migration and immune cell infiltration [41]. Subsequent replacement of cardiomyocytes with fibronectin and type III collagen develops less compliant, stiff myocardium [42]. These early biomechanical changes are sensed by cell surface integrins on cardiac fibroblasts, activating focal adhesion kinase (FAK) and downstream RhoA/ROCK signalling, which drives actin cytoskeletal remodelling in fibroblasts via the yes-associated protein (YAP) and transcriptional coactivator with PDZ-binding motif (TAZ) pathway [43,44]. Evidently, the early precipitating factor of ECM and mechanotransduction perpetuates acute cardiac fibrosis. These findings are further elucidated as the inhibition of FAK with PF-573228, an ATP-competitive small-molecular inhibitor of FAK in murine MI models significantly decreases fibrotic score and preserved partial left ventricle (LV) function [43]. Furthermore, deletion of YAP/TAZ in vivo results in reduced fibrosis, hypertrophy, and increased angiogenesis [43]. Collectively, these findings emphasise the role of mechanical stress as a causal driver of fibrosis. Thus, there is a need to develop a therapeutic intervention that focuses on the acute post-infarction period.

### 3.3. TGF-β/SMAD Signalling

The activation of the transforming growth factor-β (TGF-β)/SMAD pathway critically determines a pro-fibrotic response following AMI [45]. Physiologically, TGF-β is stored within the ECM, along with latent TGF-β binding proteins (LTBPs). Sudden accumulation of ROS initiates the proteolytic cleavage of LTBPs into active TGF-β [46]. Following liberation, TGF-β recruits the type II receptor (TβRII), which phosphorylates the type I receptor/activin receptor-like kinase 5 (TβRI/ALK5) [47]. Acting as an acute stress signal, SMAD2, SMAD3 and SMAD4 are recruited and phosphorylated, initiating fibroblast-to-myofibroblast transition at the infarct site [46]. The functional consequences of this pathway are double-edged. Early activation is essential for suppressing inflammation and stabilising the infarct scar, yet persistent signalling promotes maladaptive ECM deposition and ventricular remodelling [48]. This balance is emphasised as pre-infarction blockade via TβRII receptors amplifies inflammation and ventricular dysfunction, while post-infarct inhibition attenuates fibrosis, LV dilatation, apoptosis and progressive dilatation [49]. A study conducted by Okada et al., (2005) utilises gene therapy to suppress TGF-β signalling, critically reducing pathological fibrosis, without altering infarct size [50]. This purported mechanism of cardiac fibrosis further emphasises the need for acute therapeutic interventions to prevent the accumulation of DAMPs, particularly ROS, to negate progression to pathological cardiac fibrosis.

## 4. Augmentation of Death Signals

Rather than resolving with clearance of necrotic debris, molecular stress signals persist and intensify in the peri-infarct myocardium [38]. Sustained inflammatory cytokine exposure and ongoing metabolic stress maintain death signalling in border zone cardiomyocytes. These cardiomyocytes remain viable, yet highly vulnerable. As such, inflammatory and stress-activated pathways continue to drive acute cardiomyocyte elimination and pro-fibrotic signalling. As these death signals accumulate, fibroblast activation is sustained. Excessive extracellular matrix deposition follows, promoting progressive expansion of fibrotic tissue beyond the original infarct core [38]. With further understanding of death signal augmentation, therapeutic interventions must target the acute post-infarction window to limit maladaptive remodelling and preserve viable myocardium.

### 4.1. Type I Interferon Signalling: IFNAR-Dependent Amplification

A pro-fibrotic state is perpetuated by the cGAS–STING pathway, enabling the release of type I interferons (IFN-α/β) from injured cardiomyocytes [51]. In an autocrine fashion, IFN-α/β binds to IFNAR, causing the downstream activation of Janus kinase 1 (Jak1) and tyrosine kinase 2 (TYK2) [52]. Subsequent phosphorylation of STAT1 and STAT2 forms the ISGF3 transcription complex, driving broad transcription of interferon-stimulated genes (ISGs) [52]. Spatial transcriptomic studies further demonstrate that IFNAR1 is selectively upregulated in the peri-infarct region, where type I interferon signalling persists well beyond the acute phase of injury [53]. The formation of a pro-inflammatory environment amplifies immune cell recruitment [53]. In conjunction, sustained IFN-α/β signalling promotes increasing surface presentation of stress-inducible ligands, such as the aforementioned RAE-1 [37,53]. Critically, prolonged ISG activation extends stress ligand expression beyond the initial mtDNA-driven burst [34,35]. This increases the visibility of stressed yet potentially salvageable cardiomyocytes to cytotoxic lymphocytes, thereby promoting scar expansion and progression to HF [38]. Preclinical studies demonstrate that IFNAR1 antibody blockade enables a 30% reduction in infarct size [54]. Similarly, deletion of STING or IRF3 limits pro-inflammatory cytokine production and preserves cardiac function in mice [51]. Collectively, these findings highlight the importance of a discrete, time-limited therapeutic target to protect stressed peri-infarct cardiomyocytes and prevent adverse fibrotic remodelling.

### 4.2. Wnt/β-Catenin Signalling

Acute cardiac fibrosis is increasingly recognised as a process that is orchestrated by the reactivation of the Wnt/β-catenin pathway. This pathway remains essential during cardiac development, yet quiescent in the adult heart [55]. Following infarction, the release of inflammatory cytokines, released by mononuclear cells and mechanical ECM disturbances, prompts pathway reactivation. This is driven by the release of Wnt ligands from stressed cardiomyocytes, endothelial cells and cardiac fibroblasts [56]. Canonical Wnt ligands bind Frizzled receptors in concert with low-density lipoprotein receptor-related protein 5 and 6 (LRP5/6), inhibiting the β-catenin destruction complex [57,58]. This disruption enables the rapid cytoplasmic accumulation and nuclear translocation of β-catenin, where it associates with T-cell factor and lymphoid enhancer factor (TCF/TEF) transcriptional factors [59]. Expression of genes promoting fibroblast proliferation, myofibroblast differentiation and ECM production ensues [59]. Thus, canonical Wnt signalling integrates inflammatory signalling and mechanical stress into a profibrotic environment, which worsens acute cardiac fibrosis. Further to this, preclinical studies implicate canonical Wnt signalling as a driver of pro-fibrotic remodelling. The use of a small-molecule inhibitor, ICG-001, to inhibit β-catenin and CBP interaction mitigates downstream fibrosis and improves ejection fraction by 8.4% [60]. Similarly, pyrvinium and Frizzled receptor antagonists, which aim to prevent the inhibition of the β-catenin destruction complex, reduces infarct expansion and preserves cardiac function in rat MI models [61,62]. Collectively, these findings reinforce the notion that prolonged Wnt/β-catenin signalling stemming from early DAMP accumulation initiates a persistent profibrotic programme driving fibrotic expansion.

### 4.3. Amplifiers of TGF-β Signalling: RAAS and Endothelin-1

Although the canonical TGF-β/SMAD pathway promotes acute fibrosis following AMI, its persistence is shaped by upstream neurohormonal signalling. Among these are the renin–angiotensin–aldosterone system (RAAS) and endothelin-1 (ET-1) pathway [63,64]. Each mechanism amplifies TGF-β signalling, prolonging cellular stress and increasing progression to aberrant ECM remodelling.

Injured cardiomyocytes, immune cells and fibroblasts locally produce renin and angiotensin-converting enzyme (ACE), driving myocardial production of angiotensin II (Ang II) from angiotensinogen [65]. Ang II subsequently engages the angiotensin II type-1 receptor (AT1R) on cardiac fibroblasts, triggering downstream activation of extracellular signal-regulated kinase 1/2 (ERK1/2), p38 mitogen-activated protein kinase (p38 MAPK), and PKC pathways [66]. Substantial preclinical evidence demonstrates that Ang II-dependent cascades induce TGF-β synthesis [67]. In vitro studies emphasised the ability of Ang II to increase TGF-β1 expression in cardiac fibroblasts, myofibroblasts and cardiomyocytes [68,69]. Importantly, chronic in vitro Ang II administration further increases myocardial TGF-β1 expression, directly reinforcing the TGF-β pathway within the heart [68]. By amplifying TGF-β production and downstream SMAD2/3 activation, RAAS accelerates ECM deposition, persistent myofibroblast activation, and progressive expansion of fibrosis into the peri-infarct zone.

A second layer of amplification arises from ET-1, a potent vasoconstrictor peptide that amplifies the fibrotic effect of TGF-β [64]. TGF-β induces ET-1 expression in vascular endothelial cells and fibroblasts via SMAD signalling. This induction sustains ET-1 production, perpetuating RhoA/ROCK and ERK signalling through activation of the ET_A_ receptor [70,71]. Thus, ET-1 serves to establish a self-reinforcing profibrotic loop, manifesting as myofibroblast differentiation and increased ECM deposition [71,72]. Pharmacological inhibition of ET-1 signalling attenuates this autocrine feedback loop, thereby attenuating the downstream fibrotic consequences of TGF-β activation [73].

## 5. Cardiomyocyte Cell Death

Following the initial cardiomyocyte stress, the infarct zone transitions into a phase of continued cellular clearance and ECM deposition. However, these same pathways can extend beyond the infarct core, driving the elimination of metabolically stressed-yet-viable cardiomyocytes [74]. Within the peri-infarct border zone, innate and adaptive immune responses perpetuate a secondary phase of cardiomyocyte loss, involving non-selective cytotoxic injury, phagocytic clearance, and stress ligand-dependent killing (Table 1). Immune-mediated elimination expands the zone of death and scar formation beyond the infarct site [74,75]. Evidently, identification of accumulating DAMPs for targeted therapeutic interventions can serve to reduce acute cardiac fibrosis and mitigate progression to HF.

### 5.1. Myeloid Cells

Myeloid cells are the dominant early responders to the MI, playing a central role in shaping the inflammatory microenvironment and driving secondary cardiomyocyte injury [76,77]. Rapid recruitment is driven by the accumulation of DAMPs such as S100A8/A9 and chemokines CXCL1, CXCL2, and IL-8 from necrotic cardiomyocytes. This response promotes debris clearance, release of antimicrobial peptides and the initiation of tissue repair [24]. However, the effector functions of neutrophils and macrophages are inherently non-selective. In particular, formation of neutrophil extracellular traps (NETs) promotes microvascular obstruction and recruitment of lymphoid innate inflammatory cells, thereby expanding tissue injury and infarct size [76,77,78]. Experimental studies elucidate how suppression of neutrophil activity can significantly reduce infarct size [79,80,81]. However, this approach lacks consideration of the cardioprotective functions mediated by neutrophils, including debris clearance and the initiation of appropriate ECM remodelling [78].

In parallel with neutrophil activation, CCR2+ monocytes are recruited to the infarcted myocardium. These monocytes subsequently differentiate into inflammatory macrophages that facilitate clearance of injured cardiomyocytes through ROS generation, pro-inflammatory cytokine release and phagocytic activity [82]. Over time, macrophage phenotypes transition toward the M2-like reparative state that promotes angiogenesis and ECM deposition. However, this shift is accompanied by increased TGF-β production, linking immune-mediated clearance to the development of SMAD-induced fibrotic scar formation [20,45]. Critically, experimental strategies aimed at limiting macrophage recruitment include nanoparticle siRNA silencing and genetic CCR2 deficiency [83,84]. Both approaches demonstrate infarct mitigation through the reduction in cytokine burden and lymphocyte recruitment. Yet, monocyte blockade remains an unfavourable choice given the role of macrophages in necrotic-tissue clearance and in defining repair [82]. Neutrophil and monocyte infiltration may increase infarct size, yet suppression of the myeloid cells may compromise later myocardial healing. Therefore, therapeutic strategies must focus on the timely inhibition of DAMP and ROS accumulation to mitigate cardiomyocyte death signalling and subsequent necrosis.

### 5.2. Lymphoid Cells

Injured cardiomyocyte elimination by lymphoid immune cells centres around stress ligand-dependent cytotoxicity. Rapid upregulation of the aforementioned death signals, specifically DAMPs, PRRs and RAE-1, within the peri-infarct zone marks stressed-yet-viable cells for elimination [24,38,39]. While NK cells, NKT cells, and γδ T cells are recruited through various stress signals (Table 1), each lymphoid cell expresses NKG2D. The elimination mechanism of these cells converges as NKG2D engagement triggers innate cytotoxicity with the release of perforin and granzyme [40]. This is accompanied by the secretion of pro-inflammatory cytokines, type I interferons, TNF-α, and IL-1β, which amplify immune recruitment and cardiac fibrosis [51]. The innate response bridges into the adaptive immune response as NKG2D functions as a co-stimulatory receptor in CD8^+^ T cells. As such, T-cell receptor-dependent killing requires a lower activation threshold. Preclinical studies demonstrate NKG2D blockade attenuates fibrotic remodelling and preserves left ventricular function [40]. While focus on lymphoid cell depletion studies (Table 1) have displayed reduced infarct size [36,40,85,86,87], immunosuppression poses risks of poor tissue repair and compromising host defence [88]. Critically, the role of each immune cell represents a synchronised approach in cell elimination and progression to fibrosis. Therefore, depletion of one type of cell remains futile, as the combined function of myeloid and lymphoid cells overlaps, resulting in persistent fibrosis. Therefore, therapeutic interventions must focus on the upstream accumulation of DAMPs and death signalling from the initial ischaemic event.

**Table 1:** Role of immune cells in cardiomyocyte stress following AMI. Stress cell elimination categorised into myeloid and lymphoid branches of immunity.

Category	Molecular Target	Causal Factors	Mechanistic Summary	Key Study Findings
Myeloid stress Cell Elimination	Neutrophil influx, immune and protease mediated damage	IL-8 release: platelet, macrophage and cardiomyocytes.	Neutrophils clear debris, release ROS, elastase/MMP-9; fibroblast remodelling.	Essential for debris clearance; excessive ROS damages myocardium; ↑ fibrosis [76,77,78]
	Monocyte–macrophage recruitment and polarisation	CCR2+ monocytes → M1/M2 macrophages.	M1 amplifies inflammation; M2 resolves inflammation and secrete TGF-β, VEGF, TIMPs for repair.	Monocyte blockade; reduced cytokine burden and adverse remodelling [83,84]
Lymphoid stress Cell Elimination	Natural killer (NK) cell recruitment	DAMPs from necrotic cardiomyocytes (mtDNA, nDNA, S100A8/A9, HMGB1); IL-12/IL-15.	Early innate response; cytotoxic killing of stressed cardiomyocytes; IFN-γ secretion to enhance macrophage activity.	Mouse AMI models: NK depletion; reduced infarct inflammation; early NK activation correlates with adverse remodelling [40]
	Natural Killer T (NKT) cells	Lipid antigens via CD1d; DAMPs (mtDNA, nDNA, HMGB1); IL-12/IL-15.	Bridge innate/adaptive immunity; secrete IFN-γ, IL-4, IL-10; modulate macrophage polarisation M1 → M2.	Mouse AMI models: NKT deficiency; delayed resolution of inflammation; activation improves infarct healing [85]
	γδ T cells	Stress-induced ligands on cardiomyocytes, HMGB1, IL-1β.	Early responders; produce IL-17 and IFN-γ; recruit neutrophils; amplify inflammation.	Mouse AMI models: γδ T cell depletion; ↓ neutrophil infiltration; ↓ infarct size [86]
	CD-8 T cells	NKG2D co-stimulation; MHC I presentation of DAMPs.	Cytotoxic killing of stressed cardiomyocytes; secrete IFN-γ; influence remodelling.	Mouse AMI models: CD8^+^ depletion; reduced cardiomyocyte death, smaller infarct; chronic CD8^+^ activation; adverse remodelling [36,87]

## 6. Biomarkers of Infarct Size and Cardiac Remodelling

Clinical biomarkers reflect cardiac injury following AMI by capturing processes such as cardiomyocyte stress, acute systemic inflammation and fibrotic remodelling. In this nature, the extent of cardiac fibrosis and assessment of disease can be determined through the use of bedside tests [89,90]. Importantly, biomarker profiles can indicate the likelihood of progression to HF and increased mortality risk. In this context, biomarker data can be used to evaluate the relevance of the proposed mechanisms driving post-infarct fibrosis [89]. Critically, this approach can highlight gaps for therapeutic interventions to prevent progression to HF.

### 6.1. Clinical Markers of Cardiomyocyte Necrosis

Release of intracellular cardiomyocyte proteins serves as a direct surrogate of infarct size and peri-infarct zone stress. Clinically, the diagnosis of AMI relies on serum cardiac troponin concentrations exceeding the 99th percentile upper reference limit, defined as greater than 14 ng/L for cardiac troponin T [91]. Functionally, cardiac troponins are integral sarcomeric proteins which initiate cardiac contraction [92]. During ischaemic injury, disruption of cardiomyocyte integrity leads to an increase in serum troponin T. Acting as a DAMP, troponin T activates downstream TLR4 and NF-κB, promoting further pro-inflammatory cytokine release and local neutrophil recruitment [93]. Clinical studies have demonstrated that elevated troponin T levels correlate with larger infarct size, impaired ventricular functioning and progression to HF [94]. Specifically, patients with a serum troponin level of 25,000 pg/mL experienced a reduced LVEF of less than 40% [95]. Consequently, quantification of circulating troponin provides a valuable clinical tool for myocardial injury. It further poses value in terms of evaluating emerging therapeutic strategies for post-infarct fibrosis management.

Creatine kinase-MB (CK-MB) and lactate dehydrogenase (LDH) are less specific clinical markers of AMI, but remain clinically informative indicators of cardiomyocyte necrosis. Elevated serum levels of CK-MB and LDH, demonstrate a greater risk of post-infarct HF [96,97]. Responsible for myocardial ATP production, increasing CK-MB represents early metabolic collapse, loss of sarcolemmal integrity and the promotion of cardiomyocyte necrosis [98]. Clinically, higher peak CK-MB concentrations correlate with larger infarct size and reduced ventricular function, with peak values of 300 IU/L associated with a LVEF of below 40% [99]. Contrastingly, LDH is a non-specific marker of cellular necrosis which reflects increased anaerobic glycolysis following ischemia [96]. Consistent with this, Brigic et al. demonstrated that with each rising unit of LDH thus correlated to a 0.01% decrease in LVEF [100]. Collectively, markers of cardiomyocyte necrosis can predict infarct size, revealing strategies to quantify future potential therapeutic targets to attenuate fibrosis and improve patient prognosis.

### 6.2. Clinical Markers of Systemic Inflammation and Haemodynamic Stress

Necrosis-induced inflammation exacerbates AMI injury, as infiltrating neutrophils and monocytes accumulate and damage viable cardiomyocytes in the peri-infarct zone. As such, ischemic injury extends past the initial infarct core [101]. This injury is accompanied by a pronounced acute-phase response, characterised by elevated inflammatory reactants that burdens the peri-infarct zone. Induced by IL-6-mediated activation of hepatocytes, C-reactive protein (CRP) reflects systemic inflammation and enhanced leukocyte recruitment [102]. CRP further activates, downstream, the NF-κB pathway, which promotes pro-fibrotic cytokine release and adverse ventricular remodelling [103]. Through complement activation and leukocyte adhesion, CRP amplifies inflammatory cell infiltration, promoting increased fibroblast-to-myofibroblast differentiation within the infarct site [76]. Consequently, elevated CRP levels are associated with larger peri-infarct zones, increasing susceptibility to HF.

In a similar vein, B-type natriuretic peptide (BNP) is a marker of haemodynamic stress that correlates with infarct size post AMI [104]. BNP is secreted by ventricular myocardium in response to increased ventricular wall stretch and pressure overload. Although BNP exerts compensatory natriuretic and vasodilatory effects, sustained elevation post MI reflects maladaptive ventricular remodelling [104,105]. Persistent haemodynamic stress activates pro-fibrotic pathways, including TGF-β/SMAD signalling and YAP/TAZ mechanotransduction, which collectively promote excessive ECM deposition and myocardial stiffening [42,48]. This is demonstrated by Omland et al. (1996), who showed that elevated BNP concentrations are associated with larger infarct size, reduced LVEF and increased risk for progression to HF [106]. Together, the biomarkers CRP and BNP provide complementary insight into inflammation and haemodynamic stressors post MI. Critically, both clinical biomarkers can serve as predictive values of infarct size, acting as final outcomes for the development of therapeutic interventions.

### 6.3. Fibrosis Timeline: Therapeutic Window

Understanding the timeline of different pro-fibrotic mechanisms can elucidate potential therapeutic approaches. The evolution of post-infarct fibrosis can therefore be characterised into hyperacute (minutes to hours), acute (hours–days), sub-acute (days to weeks) and beyond acute (weeks to months). Within the hyperacute stage, necrotic cardiomyocytes release DAMPs including mtDNA, HMGB1, ATP and S100A8/A9 [17,25,38]. In this phase, activation of PRRs, including TLR2 and TLR4, initiates downstream NF-κB and NLRP3 signalling [16,17,24,26,30,31]. In parallel, mitochondrial destabilisation activates the cGAS-STING axis, promoting type I interferon signalling and stress ligand expression of RAE-1 [34,35,36,37,38,39,40]. These early events align with clinically rapid increases in cardiac troponin and CK-MB, which reflect cardiomyocyte membrane disruption [91,92,93,94,95]. This critically correlates with infarct size and peri-infarct zone stress. These pathways define a narrow early therapeutic window in which attenuation of DAMP accumulation and innate immune activation may preserve viable myocardium.

As injury progresses into further into the acute phase, amplification of inflammatory and profibrotic signalling becomes dominant. This occurs simultaneously with increased systemic markers of inflammatory and haemodynamic stress. Sustained IFNAR-dependent signalling promotes immune recruitment [51,52,53,54], while reactivation of canonical Wnt/β-catenin promotes fibroblast proliferation and ECM synthesis [55,56,57,58,59,60,61,62]. Concurrently, neurohormonal activation through RAAS and endothelin-1 reinforces TGF-β/SMAD signalling, further supporting fibroblast-to-myofibroblast transition and progressive ECM deposition [45,46,47,48,49,50,63,64,65,66,67,68,69,70,71,72,73]. Clinically, this aligns with elevations in CRP and BNP, indicating continued inflammatory activation and ventricular wall stress, which represents increased HF risk [102,103,104,105,106]. The beyond acute phase emphasises the consolidation of scar tissue and ventricular stiffening [42]. Therapeutic efficacy following AMI likely depends on pathway-specific timing. Therefore, interventions focussing on the acute stage of DAMP-driven immune activation offers the greatest potential to preserve peri-infarct myocardium [16,17]. Overall, attempting to prevent infarct injury during the hyperacute stage attenuates further activation of pro-inflammatory pathways, reducing the risk of HF.

## 7. Proposed Strategies in Development

PCI remains the current gold standard for AMI. Yet this strategy alone does not provide adequate protection against cardiomyocyte stress and necrosis, which potentially exacerbates infarct size [10]. Given the multitude of molecular pathways implicated in cardiac fibrosis, numerous potential targets have been identified. Among these approaches to attenuating cardiac fibrosis, non-specific cell-targeted therapy and systemic anti-inflammation remain the most prominent. Due to the substantial number of clinical studies investigating these strategies, this section will focus on human clinical trials. Centrally, existing therapies and their efficacy in limiting post-infarct cardiac fibrosis will be explored with an emphasis on acute management. A summary of selected clinical trials and therapeutic targets is presented in Table 2.

### 7.1. Cell-Targeted Therapies

The mitigation of cardiomyocyte injury following infarct requires targeted interventions to address haemodynamic overload, oxidative stress and necrosis, which drive infarct expansion and adverse remodelling [3]. Novel approaches suggest that early administration of interventions within the peri-reperfusion window is crucial to maximising cardio protection [107]. As summarised in Table 2, clinical trials have focussed on reducing cardiac load through β-adrenergic receptor blockade [108,109], attenuation of cardiomyocyte apoptosis through mitochondrial stabilisation [110,111,112,113] and reductions in platelet and leukocyte adhesion within microvasculature [114,115,116,117]. Further to this, use of nitric oxide (NO) and GLP-1 as agents to reduce ROS formation within cardiomyocytes has been investigated [118,119,120,121]. This limited translational efficacy highlights the rapid onset of irreversible cardiomyocyte death following AMI. Strategies focussing on mitochondrial stabilisation fall short as ATP depletion, mitochondrial membrane instability, Ca^2+^ overload ensue [11]. Following mitochondrial degradation, stabilising interventions such as cyclosporin and MTP-131 fail to mitigate infarct size [110,111,112,113]. Similarly, β-adrenergic receptor blockade outcomes rely on pre-reperfusion delivery to attenuate metabolic stress, such as mitochondrial ATP depletion, ROS generation and lipolysis within the peri-infarct border zone [109]. Additionally, interventions focussing on ROS pathways are constrained by redundancy in ROS generation pathways. An inability to prevent the aforementioned DAMPs and PRR signalling results in a continued production of pro-inflammatory pathways [16]. Despite strong mechanistic rationales, each study has generally reported negligible change regarding infarct size (Table 2). The variability in infarct size outcomes remains strongly dependent on system delay and timing relative to reperfusion, emphasising that even theoretically protective agents may fail if administered sub-optimally. Overall, the redundancy of cell death and inflammatory pathways limits the impact of cell-targeted interventions. Critically, parallel mechanisms continue to drive necrosis and cardiac fibrosis despite therapy.

### 7.2. Systemic Anti-Inflammatory Therapies

In a similar fashion, attenuating inflammation following AMI has been explored thoroughly to mitigate acute fibrosis and later adverse remodelling. Inflammation is essential for the clearance of necrotic debris and initiation of tissue repair. However, when prolonged stressed-yet-viable immune cells undergo necrosis and promote further scar formation. Therefore, the therapeutic approaches have sought to modulate inflammatory signalling, specifically the inhibition of cytokine pathways and innate immune activation. Currently, past clinical trials have explored the blockade of IL-6 and IL-1 receptors [122,123], antibody CD20 B-cell depletion [124] and C5 complement inhibition [125]. Despite promising preclinical data, translation to clinical benefit has been inconsistent. This is a result of systemic inflammatory pathways possessing substantial redundancy, with varying cytokines and immune cells capable of compensating when one pathway is inhibited [126]. Specifically, these interventions act downstream of early innate immune sensing pathways. Without management of TLR, NLRP3 inflammasome and cGAS-STING activation, stress ligands such as RAE-1 and lymphocyte recruitment continue within the peri-infarct zone [16,31]. Similarly, inhibition of individual cytokines promotes compensatory activation of parallel inflammatory cascades such as TNF-α, MCP-1 and type I interferon signalling [20,26]. Thus, targeting specific inflammatory mediators has produced modest reductions in infarct size or clinical outcomes. Immunosuppression further poses the risks of poor tissue repair and susceptibility to infection [88]. Therefore, targeted therapies must focus on inhibiting early-onset inflammation directly post AMI.

**Table 2:** Current Clinical Studies Exploring the Mitigation of Infarct and Cardiomyocyte Stress. Novel post-acute myocardial infarction (AMI) strategies categorised into cell-targeted and anti-inflammatory therapy.

Category	Functional Target	Therapeutic Agent	Duration	Status	Key Study Findings
Cell-Targeted Therapy	β-adrenergic receptor blockade	Metoprolol (intravenous)	Intravenously before PCI and daily oral intake	Concluded	20% reduction in infarct size 5–7 days after STEMI. Increased LVEF vs. control (~2.67% adjusted difference) [108]. No effect on infarct size compared to placebo [109].
	Inhibit cyclophilin D and mitochondrial permeability transition pore	Cyclosporine (intravenous)	Immediately before PCI	Concluded	No significant difference in the safety profile, clinical outcomes, and LV remodelling at 1 year [110,111,112].
	Cardiolipin in cardiomyocyte mitochondria	MTP-131 (intravenous)	Immediately before PCI	Concluded	No effect on MI size compared to placebo (72 h AUC total CK) [113].
	Glycoprotein IIa/IIIb on platelets (anti-platelet)	Abciximab (intravenous)	Direct injection into infarct site: 0.25 mg/kg with bivalirudin as procedural anticoagulant	Concluded	Reduced MI size at 30-day compared to placebo [114]. Control: Median: 15.1%; interquartile range Treatment: 17.9%; *p* = 0.03
	Inhibition of vascular endothelial cadherin. Competitive antagonist of E1 fibrin fragment.	FX06 (Intravenous)	Immediately before PCI	Concluded	Significant 58% reduction in infarct size compared to placebo at 5 days. No difference in scar size after 4 months [115].
	Reduce endothelial cell swelling, inhibit leukocyte adhesion and increase oxygen delivery	Supersaturated oxygen (Intracoronary infusion)	90 min intracoronary infusion before PCI	Progressed to IC-HOT study and approved by FDA	Smaller MI size (26.5% in experiment group vs. 20% in control) at 5–7 days [116]. One-year rate 0% of all-cause death or new-onset heart failure compared to 12.3% in control [117].
	GLP-1 receptor agonist	Exenatide (intravenous)	15 min before PCI and 6 h after	Concluded	For system delay < 132 min (first medical contact to first balloon), 30% reduction in infarct size at 3 months when compared to placebo [118].
	GLP-1 receptor agonist	Liraglutide (subcutaneous)	Before PCI and maintained for 7 days after PCI	Concluded	A 29% reduction in infarct size (15 ± 12 in liraglutide group versus 21 ± 15 g in placebo group; *p* = 0.05) and higher cardiomyocyte salvage index of 17% [119].
	cGMP/PKG signalling cascade	Sodium nitrite (intravenous)	Immediately before PCI	Concluded	No effect on MI size compared to placebo (72 h AUC total CK) [120].
	Reactive oxygen species scavenger in cardiomyocytes	High-dose N-acetylcysteine (29 g over 2 days), low-dose nitro-glycerine (7.2 mg over 2 days)	48 h after PCI	Concluded	A 5.5% reduction in CMR-assessed MI size (7 days) compared to placebo. 100% increase in myocardial salvage index [121].
Anti- Inflammatory Therapies	IL-6 receptor blockade	Tocilizumab (monoclonal antibody, intravenous)	A single dose of 280 mg promptly after admission (before PCI)	Concluded	Increased myocardial salvage index compared to placebo. No significant difference in infarct size compared to placebo [122].
	IL-1 receptor antagonist	Anakinra (intravenous)	14 days (100 mg/day)	Concluded	No significant effect on infarct size compared to placebo [123].
	Anti-CD20 antibody-mediated depletion of B-cells	Rituximab (monoclonal anti-CD20 antibody, intravenous)	A single dose within 48 h of symptom onset	Progressed to RITA MI-2 trial (ongoing Phase-IIb)	Phase-I/IIa completed. 96% depletion of B-cells within 30 min, and no adverse events. Clinical efficacy yet to be tested in RITA MI-2 trial [124].
	Inhibits complement C5 acute phase protein	Pexelizumab (intravenous)	2 mg/kg bolus before PCI followed by 0.05 mg/kg hourly for 1 day	Concluded	No significant effect on infarct size compared to placebo [125].

## 8. Emergent Therapeutic Strategies

Despite growing interest in antifibrotic therapies, several mechanisms that may be relevant to the treatment of cardiac fibrosis remain insufficiently explored. Thus, identifying interventions that act during the acute phase remains critical to limit cardiac fibrosis after injury. Newer methods of regulation, such as the use of long non-coding RNA (lncRNA) networks, immune stromal interaction and biotechnological strategies, have remained incompletely defined in the context of AMI therapies. Further study is required to define the role of these uncharacterised mechanisms in fibroblast activation and ECM remodelling, and their viability as antifibrotic targets. Nevertheless, they provide a useful foundation for subsequent validation and translational investigation.

The use of lncRNAs has emerged as a potential strategy to attenuate cardiac fibrosis. Several lncRNAs, such as Cfast and Wisper, are enriched within cardiac fibroblasts and play critical roles in regulating fibrosis. Cfast is a cardiac fibroblast-enriched transcript that is upregulated following MI and affects ECM remodelling following acute cardiac injury [127]. Zhang et al. have shown that depletion of Cfast during or after AMI can reduce fibrosis and improve heart function in mouse models [127]. Similarly, Wisper is a polyadenylated and multiexonic CF-enriched transcript conserved in the human heart and controls fibroblast proliferation, migration, and survival. Its levels in the human heart correlate with collagen deposition and fibrosis severity [128]. Inhibiting Wisper expression in vivo significantly reduces fibroblast proliferation and thinning of myocardial walls after MI [128]. These studies suggest that targeting fibroblast-specific lncRNAs could be a promising way to prevent pathological fibrosis and HF risk.

MicroRNAs (miRNAs) are a conserved class of small 22-nucleotide noncoding RNAs that post-transcriptionally repress target gene expression and regulate key processes underlying cardiac pathophysiology [129]. Multiple studies have demonstrated that miRNAs modulate cardiac fibrosis by targeting key regulatory factors and signalling pathways. miR-24-3p is a multifunctional microRNA that modulates gene expression at the post-transcriptional level, playing a key role in cell proliferation, differentiation, apoptosis, and pathological development [130]. Zhang et al. has shown that overexpression of miR-24-3p reduces cardiac fibrosis in the left ventricular tissues in transverse aortic constriction (TAC) mice, accompanied with decreased expression of collagen 1,2,3 and anti-smooth muscle antibody concentrations [127]. More importantly, increased miR-24-3p levels also seemed to recover cardiac function upon miR-24-3p viral vector administration [127]. Collectively, miR-24-3p emerges as a promising novel target for anti-fibrotic therapy in cardiac fibrosis.

Another compelling and contrasting candidate is microRNA-21 (miR-21), a highly expressed regulator of cardiac fibrosis that promotes fibroblast survival, proliferation, and differentiation into collagen-producing myofibroblasts, largely through TGF-β-dependent signalling. miR-21 is markedly upregulated in cardiac fibroblasts following AMI, perpetuating increasing HF risk [131]. As such, miR-21 has emerged as both a therapeutic target and a biomarker [132], with its inhibition shown to attenuate pathological remodelling. Thum et al. (2008) [131] demonstrated that upregulation of miR-21 enhances ERK–MAPK signalling, thereby promoting fibroblast proliferation and the development of cardiac fibrosis. Conversely, silencing of miR-21 attenuated ERK–MAPK pathway activation, resulting in reduced fibrotic remodelling and improved cardiac function [131]. In multiple preclinical models, inhibition of miR-21 with antagomirs or genetic silencing has been shown to reduce pathological cardiac remodelling and fibrosis while improving cardiac function.

Direct cardiac reprogramming has emerged as a promising regenerative strategy for heart failure, aiming to restore lost myocardium by converting resident non-myocytes, particularly cardiac fibroblasts, into induced cardiomyocyte-like cells. This approach bypasses a pluripotent intermediate state and instead relies on defined transcription factors, microRNAs, or small molecules to activate cardiomyocyte gene programmes [133]. In preclinical models of cardiac injury, direct reprogramming has been shown to reduce fibrotic burden, enhance myocardial contractility, and improve cardiac function. This approach by Tani et al. is founded on the ability of cardiac reprogramming to suppress fibroblastic gene expression in chronic myocardial infarction by shifting profibrotic fibroblasts toward a quiescent antifibrotic state [134]. By targeting endogenous cell populations within the injured heart, this strategy offers a potential means of myocardial regeneration while avoiding challenges associated with cell transplantation, such as poor engraftment and immune rejection [134].

Using the same set of transcription factors MEF2C, GATA4, TBX5, and HAND2 (MGTH), Song et al. demonstrated that adult mouse tail-tip fibroblasts and cardiac fibroblasts could be converted into spontaneously contracting cardiomyocyte-like cells in vitro [135]. When these factors were ectopically expressed in proliferating non-cardiomyocytes in vivo, the cells acquired functional cardiomyocyte-like properties, which were accompanied by improved cardiac performance and a reduction in maladaptive ventricular remodelling following MI.

## 9. Future Directions: Proposed Therapeutic Interventions

While the role of CD8+ T cells in the aetiology of cardiac fibrosis has been largely delineated, further mechanistic insight may be gained by shifting focus toward their interaction with NKG2D stress-induced surface ligands, specifically with RAE-1 [37]. This crosstalk between NKG2D and RAE-1 is sufficient to activate CD8+ T, NKT and NK cells, triggering innate killing responses, amplifying tissue injury and promoting fibrotic remodelling [40]. Following AMI, acute ischaemic cardiomyocyte stress promotes increased reactive oxygen species production and cytoplasmic DNA release, thereby engaging the cGAS–STING signalling axis. This STING pathway activation causes the stressed cardiomyocytes to express RAE-1 on their surface [34,35]. RAE-1 serves as an activating ligand for the NKG2D receptors expressed on cytotoxic CD8^+^ T cells within the cardiac tissue, leading to perforin release and cardiomyocyte apoptosis [40]. This ultimately results in cardiac fibrosis and stiffening of the heart muscle.

In 2019, Matsumoto et al. showed that in vivo administration of RAE-1 neutralising antibody via IP injection after MI blocks the RAE-1–NKG2D axis, significantly reducing the frequency of apoptotic cardiomyocytes, accompanied by the suppression of cardiac fibrosis in murine models [40]. This approach demonstrated promising efficacy in attenuating cardiac fibrosis; however, its mode of administration lacks tissue specificity and may result in off-target effects.

An alternative strategy that offers greater targeting precision and mechanistic specificity, while achieving comparable antifibrotic efficacy, may represent a more effective therapeutic approach. Given the central role of RAE-1 in early stress and immune signalling, nanoparticles that selectively target RAE-1 may offer an efficacious novel therapeutic approach. Experimental blockade of the RAE-1–NKG2D axis reduces cardiomyocyte apoptosis and suppresses cardiac fibrosis [40]. Although RAE-1 studies remain limited to experimental systems, translational relevance is supported by investigations of its human homologue, the MHC class I polypeptide-related sequence A (MICA). In cardiac transplantation, elevated circulating serum MICA has been associated with adverse transplant outcomes. This activation of DNA damage and stress-induced activation of NKG2D critically contributes to cardiomyopathy [33]. Consequently, this suggests that RAE-1 is not only a marker of injury but also a functional driver of cardiomyocyte death and pathological remodelling. Overall, this highlights RAE-1 as a potential therapeutic target in mitigating acute cardiac fibrosis.

Nanoparticles continue to offer transient, cell-targeted delivery of miRNA, making them suited to the narrow therapeutic window post-AMI. This may achieve short-term modulation of stress-induced signalling, but may limit maladaptive cardiac remodelling. Critically, targeted lipid nanoparticle delivery has demonstrated efficacy in experimental cardiac disease models. A study conducted by Hou et al., demonstrated that targeted lipid nanoparticles achieved efficient cardiomyocyte transfection in vivo following systemic administration [136]. Similarly, Ou et al. demonstrated that ECM–lipid nanoparticle composites enabled rapid site-specific siRNA delivery following MI [137,138]. Furthermore, nanoparticle delivery systems have been proven to be able to selectively deliver miRNA to the infarct site. Kanki et al. (2011) demonstrated selective accumulation of miRNA-loaded nanoparticles in ischaemic myocardium, enabling spatially restricted and targeted interventions [139]. As RAE-1 contributes to immune responses against infection, a transient, early post-infarction delivery of RAE-1-silencing nanoparticles could emerge as a highly effective therapeutic option to systemic immunity.

## 10. Conclusions

Cardiac fibrosis remains the major determinant for progression to HF after AMI. Critically, the mechanisms of fibrosis remain not fully understood. Current therapies centre around reperfusion, which does not prevent the inflammatory, mechanical and profibrotic signals that extend injury beyond the infarct core. From the upregulation and augmentation of death signals to cell elimination, the fibrotic process possesses many biological redundancies. Therefore, inhibition of one area does not fully mitigate inflammation and further ECM deposition. Future progress will thus depend on interventions delivered early to preserve stressed but viable myocardium to prevent maladaptive scar formation, to increase patient quality of life and reduce the burden of HF.

## Data Availability

No new data were created or analyzed in this study. Data sharing is not applicable to this article.

## Abbreviations

The following abbreviations are used in this manuscript:

AMI	Acute myocardial infarct
HF	Heart failure
PCI	Percutaneous coronary intervention
ECM	Extracellular matrix
ATP	Adenosine triphosphate
ROS	Reactive oxygen species
DNA	Deoxyribonucleic acid
NLRP3	NLR family pyrin domain-containing 3
NF-κB	Nuclear factor- κB
GLP-1	Glucagon-like-peptide-1
PKC	Protein kinase C
PI3K/AKT	Phosphoinositide 3-kinase-protein kinase B pathway
DAMPs	Damage-associated molecular patterns
mtDNA	Mitochondrial DNA
PRRs	Pattern-recognition receptors
TLR	Toll-like receptor
LVEF	Left ventricular ejection fraction
cGAS	Cyclic GMP-AMP synthase
STING	Stimulator of Interferon Genes
MCP-1	Monocyte chemoattractant protein-1
IFNAR	Interferon-α and β receptor subunit
RAE-1	Retinoic acid early Inducible-1
NKG2D	Natural killer group 2, member D
NK cells	Natural killer cells
MMPs	Matrix metalloproteinases
FAK	Focal adhesion kinase
YAP	Yes-associated protein
LV	Left ventricle
TAZ	Transcriptional coactivator with PDZ-binding motif
TGF-β	Transforming growth factor-β
LTBPs	Latent TGF-β binding proteins
T βRI/ALK5	Type I receptor/activin receptor-like kinase 5
Jak1	Janus kinase 1
STAT	Signal transducers and activators of transcription
TCF/TEF	T-cell factor and lymphoid enhancer factor
RAAS	Renin–angiotensin–aldosterone system
ET-1	Endothelin-1
AT1R	Angiotensin II type-1 receptor
Ang II	Angiotensin II
ERK1/2	Extracellular signal-regulated kinase ½
MAPK	Mitogen-activated protein kinase
CK-MB	Creatine kinase-MB
LDH	Lactate dehydrogenase
BNP	B-natriuretic peptide
CRP	C-reactive protein
NO	Nitric oxide
lncRNA	Long non-coding RNA
miRNA	MicroRNA
TAC	Transverse aortic constriction
MEF2C	Myocyte-specific enhancer factor 2C
GATA4	GATA-binding protein-4
TBX5	T-box transcription factor 5
HAND2	Heart and neural crest derivatives expressed 2
